# Editorial: Hot topic: excited state processes in biomolecules

**DOI:** 10.3389/fchem.2024.1467074

**Published:** 2024-07-31

**Authors:** Chong Fang, Nadia Rega, Malgorzata Biczysko

**Affiliations:** ^1^ Department of Chemistry, Oregon State University, Corvallis, OR, United States; ^2^ Department of Chemical Sciences, University of Napoli Federico II, Napoli, Italy; ^3^ College of Sciences, Shanghai University, Shanghai, China

**Keywords:** excited-state properties, relaxation pathways, molecular spectroscopy, quantum chemical calculations, photophysics and photochemistry, light-induced phenomena, structural dynamics, structure-function relationships

This feature collection of papers for the Research Topic “*Hot Topic: Excited State Processes in Biomolecules*” covers a broad range of research on recent advances in experimental and computational studies of excited state processes in biomolecules. The papers demonstrate a variety of state-of-the-art experimental and computational approaches to delineate light-induced transient processes in organic/biomolecules and are of broad interest to researchers working at the interface of photophysics and photochemistry, photobiology, spectroscopy, bioimaging, biomimetics, and life sciences in general.

One of the central motivations for excited-state studies is the elucidation of sunlight’s effects on Earth. This includes both positive and negative aspects, with the latter being potential UV-induced photodamage of biomolecules and possible alleviation pathways via photoprotective processes such as those occurring in DNA (Martínez Fernández et al., 2022). Meanwhile, life also depends on light-harvesting processes of photosynthetic organisms including plants, algae, and some bacteria, wherein their apparent electronic absorption peak separation by over 100 nm is intriguing for the underlying dominant chromophores of chlorophyll *a* (Chl) versus bacteriochlorophyll *a* (BChl). In a highly systematic investigation employing the absorption and fluorescence excitation anisotropy spectroscopy on 17 core complexes and 16 peripheral complexes (from wild type to engineered, and detergent-purified to membrane-embedded for valuable contrasts) across sulfur and non-sulfur purple bacteria (Timpmann et al.), a robust linear correlation between the excitation bandwidth and the lowest-energy exciton Q_y_ absorption band maximum was found particularly at low temperature (4.5 K). Complementary techniques from circular dichroism (CD) to hole-burning were also used to support the band assignment. This result reveals that the complexes with broader bandwidths and stronger coupled excitons are prone to absorb redder light, consistent with the particle-in-a-box quantum principle. Such a useful linear relationship could inspire future sustainable energy strategies and devices that can better utilize sunlight for energy production and transfer at elevated temperatures.

Since intersystem crossing (ISC) represents another excited-state process with strong implications for triplet state and thermally activated delayed fluorescence (TADF) for materials applications (Hirata et al., 2015; Yonemoto et al., 2020), a stimulating report on the significance of the inverted singlet-triplet gap (STG; negative here means the triplet state lies higher than the singlet state) provides a critical evaluation of STG origin with the cutting-edge high-level *ab initio* methods such as the third-order algebraic diagrammatic construction [ADC (3)] and coupled-cluster with singles and doubles [ΔCCSD(T)] on heptazines with fused aromatic rings and heteroatoms like nitrogen or boron (Dreuw and Hoffmann). These insightful results substantiate the importance of an accurate description/modeling of the higher-order (at least third-order) electron correlation with a suitably extended basis set (such as cc-pVTZ) and theory level, aided by exciton analysis for spatial correlation/entanglement of the hole and electron within the singlet and triplet excitons. Practical applications for the near-zero STGs with practically degenerate S_1_ and T_1_ states include the rational design and development of efficient organic light-emitting diodes (OLEDs) and functional materials.

To better exploit the power of spectroscopic theoretical methodologies (Barone et al., 2021), density functional theory (DFT) and time-dependent (TD)-DFT-based computations were used to predict the electronic absorption and emission spectra including the inherent band-shape due to pertinent vibrational effects in a perspective article (Li et al.) using novel fluorescent dyes called viologens as a test case. The focus on the readily available optical spectra and economical DFT calculations allows for deeper insights into the structural factors leading to discernible spectral patterns for a wide array of organic and inorganic dyes with broad applications (e.g., redox reagents, imaging and/or ion sensors, molecular electronics, solar energy conversion and storage). In particular, the explicit inclusion of molecular vibrations in the excited electronic states (for an accurate modeling of vibronic transitions) can increase the simulation relevance with accuracy. The demonstration for semi-rigid or moderately flexible systems could be extended to more flexible ones by incorporating advanced methods from wavepacket dynamics, effective anharmonic treatments to a combination of static and dynamic computations for a comprehensive simulation of the diverse excited-state phenomena of interest.

Among all the light-sensitive biomolecules, photoconvertible fluorescent proteins (pcFPs) belong to a group of genetically-encodable luminous biomarkers for sophisticated bioimaging, which can enable the visualization of cellular structures beyond the diffraction limit due to their ability to change emission color following light activation (Adam et al., 2008; Bourgeois and Adam, 2012; Subach and Verkhusha, 2012; Nienhaus and Nienhaus, 2014). The least-evolved ancestor (LEA) is a unique pcFP engineered via ancestral gene reconstruction to represent the evolutionary node for FPs to acquire the color-changing ability (Kim et al., 2013; Kim et al., 2015). Dual illumination under ambient light may have been preferentially evolved to accelerate the LEA photoconversion and take advantage of the sunlight spectrum (Krueger et al., 2020). In a combined steady-state and ultrafast spectroscopy, quantum calculations, protein engineering, and X-ray crystallography work, a set of five related FPs with varying photoconversion and photoswitching efficiencies were studied in electronic and vibrational domains to reveal the fluorescence modulation mechanisms (Krueger et al.). In particular, noncanonical amino acid (ncAA) incorporation produced a methyl-histidine chromophore derivative of LEA that showcases the enhanced photoswitching but a greatly reduced photoconversion efficiency versus the parent FP. The rational engineering of chromophores and local environment residues via ncAA is a promising area of research to realize substantial progress by fine-tuning the photophysical and photochemical properties of FPs. Both transient dynamics and local environment of the initial fluorescent state with a *cis* chromophore need to be considered when evaluating photoswitching in addition to the photoswitched *off* state with a *trans*-like chromophore, echoing a recent report about varying hydrogen-bonding interactions of dynamic chromophores in related pcFPs (De Zitter et al., 2020). Importantly, femtosecond stimulated Raman spectroscopy (FSRS) in the excited state (Fang et al., 2009; Fang and Tang, 2020) revealed key vibrational motions of a LEA mutant, LEA-A69T, indicating that the photoswitching process is inhibited by a π-stacked histidine ring near the chromophore which likely hinders the *cis*-to-*trans* isomerization via sterics and electrostatic interactions. Such molecular movies of biomolecules in action are expected to power the bottom-up design and engineering of versatile bioprobes and biosensors with targeted functions.

From this comprehensive line of inquiries deciphering intrinsically competitive excited-state pathways of biomolecules across their absorption, emission, internal conversion and intersystem crossing, we can appreciate the increasingly accurate predictive power of an effective feedback loop established between vibrant and collaborative organic chemists/protein engineers (“makers”) and biophysical chemists/spectroscopists/theoreticians (“analyzers”). Excited-state processes essentially power everything on Earth starting from the origin of life (abiogenesis), and we hope this Hot Topic in both *Front. Phys*. and *Front. Chem* (https://www.frontiersin.org/research-topics/50676/hot-topic-excited-state-processes-in-biomolecules/articles) has presented in one place some exciting advances about biomolecular excited-state processes and will inspire future innovations and breakthroughs across disciplines to promote a more sustainable and healthy world.
